# R and S enantiomers of CBD3063, a Ca_V_2.2 N-type calcium channel modulator, alleviate capsaicin-induced inflammatory pain

**DOI:** 10.1016/j.ynpai.2025.100185

**Published:** 2025-05-16

**Authors:** Santiago Loya-López, Erick J. Rodríguez-Palma, Aida Calderón-Rivera, Kimberly Gomez, Samantha Perez-Miller, Rajesh Khanna

**Affiliations:** a Department of Pharmacology and Therapeutics, College of Medicine, University of Florida, Gainesville, FL, USA; b Center for Advanced Pain Therapeutics and Research (CAPTOR), College of Medicine, University of Florida, Gainesville, FL, USA; c McKnight Brain Institute, College of Medicine, University of Florida, Gainesville, FL, USA

**Keywords:** CaV2.2, CRMP2, Peptidomimetic, Enantiomers, Inflammatory pain

## Abstract

N-type voltage-gated calcium channels (Ca_V_2.2) play a pivotal role in pain signaling, rendering them promising targets for pain treatment. However, direct blockers of Ca_V_2.2 have demonstrated limited efficacy due to adverse side effects and inadequate blood–brain barrier penetration. In previous work, we developed CBD3063, a small molecule peptidomimetic that disrupts the Ca_V_2.2-CRMP2 (collapsin response mediator protein 2) interaction, resulting in a reduction of Ca_V_2.2 currents and pain relief without side effects. In this study, we investigated the individual contributions of the (R) and (S) enantiomers of CBD3063 to its pharmacological effects. Whole-cell patch-clamp recordings from mouse dorsal root ganglion (DRG) sensory neurons indicated that the (S) and (R) enantiomers reduced Ca_V_2.2 currents. Furthermore, racemic CBD3063 and the (S) enantiomer exhibited antinociceptive effects in the capsaicin-induced model of inflammatory pain. These findings suggest that the (S) and (R) enantiomers contribute to the therapeutic effects of CBD3063.

## Highlights

• Both (S) CBD3063 and (R) CBD3063 similarly reduce N-type Ca2 + currents.
• The (S) enantiomer shifts Ca2 + channel inactivation leftward.
• Racemic CBD3063 shows antinociceptive effects in vivo.
• Both (S) CBD3063 and (R) CBD3063 contribute to CBD3063′s antinociceptive properties.

## 1 Introduction

N-type voltage-gated calcium channels (Ca_V_2.2) are crucial transmembrane heteromultimeric complexes involved in pain signaling. These channels are highly expressed in dorsal root ganglia (DRG) and the spinal dorsal horn (Nieto-Rostro et al., 2018), facilitating calcium influx and glutamate release from sensory neurons. Due to their significant role in pain conditions, CaV2.2 channels are key therapeutic targets for treating both neuropathic (Schroeder et al., 2006, Khanna et al., 2019) and inflammatory pain (Cizkova et al., 2002, Salib et al., 2024).

Over the past few decades, several direct blockers of Ca_V_2.2 channels, such Gabapentin (Hendrich et al., 2008), Pregabalin (Bauer et al., 2009) and Prialt (McGivern, 2007) have been developed to treat chronic pain. Despite their potential, these drugs have faced significant limitations, including serious side effects (Finnerup et al., 2005, Evoy et al., 2021) and difficulties in administration (Staats et al., 2004), which have restricted their success. Newer approaches, for instance the recently reported compound, C2230, shows promise as a state- and use-dependent Ca_V_2.2 blocker, effectively mitigating pain behaviors across various models without the adverse effects associated with earlier drug (Tang et al., 2024).

**Fig. 1:**
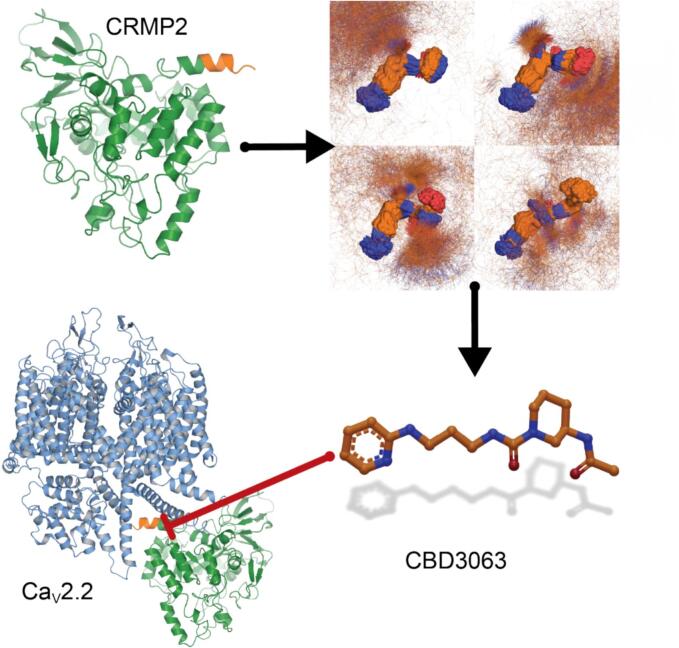
CBD3063 discovery. Clockwise from top left: Ribbon diagram of CRMP2 structure (PDB 5MKV (Zheng et al., 2018) with location of the CBD3 peptide in orange. Clustered results of molecular dynamics simulations of the CBD3 peptide used to identify the anchor motif used for pharmacophore screen that led to identification of CBD3063, which inhibits the interaction between CRMP2 and Ca_V_2.2 (Gomez et al., 2023). Ca_V_2.2 α-subunit shown as ribbons (PDB 7MIY (Gao et al., 2021).

We have advanced an alternative approach of indirectly targeting Ca_V_2.2 channels with auxiliary proteins, including the Ca_V_β subunit (Ran et al., 2021, Khanna et al., 2019) and the axonal collapsin response mediator protein 2 (CRMP2) (Chew and Khanna, 2018). We reported that CRMP2 interacts with Ca_V_2.2 channels, regulating their function and trafficking (Khanna et al., 2019, Chi et al., 2009, Brittain et al., 2009) in both central nervous system (Brittain et al., 2009) and DRG neurons (Chi et al., 2009). Furthermore, these studies led us to identify a 15-amino acid peptide sequence from CRMP2, designated CBD3 (calcium binding domain 3), which interferedb with the CaV2.2-CRMP2 interaction (Brittain et al., 2011), resulting in a reduction of N-type calcium, glutamate release and nociceptive behaviors (Brittain et al., 2011, Wilson et al., 2011). Further work led us to pinpoint key residues in CBD3 essential for its antinociceptive activity (Moutal et al., 2018, Perez-Miller et al., 2024). Based on these studies, we recently reported a first-in-class small molecule peptidomimetic, called CBD3063 ([(3R)‐3‐acetamido‐N‐[3‐(pyridin‐2‐ylamino) propyl] piperidine‐1‐carboxamide) (Fig. 1), which: 1) disrupted the Ca_V_2.2-CRMP2 interaction, 2) specifically inhibited the activity of N-type calcium channels, and 3) alleviated neuropathic and inflammatory pain via multiple routes of administration in male and female rats and mice without eliciting sensory, sedative, affective or cognitive side effects (Gomez et al., 2023, Allen et al., 2024). Collectively, these findings validate that indirect modulation of Ca_V_2.2 channels offers a novel approach for chronic pain treatment.

CBD3063, a racemic mixture, aligns with most marketed pharmaceuticals with 88 % being administered as racemic mixtures (Singh et al., 2020) and 56 % being chiral in nature. In recent years, medicinal chemistry has focused on using enantiomerically pure substances to reduce side effects associated with inactive enantiomers (Coelho et al., 2021). A well-known example is thalidomide, where (R) enantiomer acted as a sedative and was used to treat nausea in pregnant women, while the (S) enantiomer was found to cause serious birth defects (Blaschke et al., 1979). Another example is the clinically used anti-epileptic drug (R)-lacosamide (Vimpat®), which targets a sodium channel (Wang et al., 2010) while its inactive enantiomer, (S)-lacosamide, is inactive (Moutal et al., 2016). Considering the development and regulatory hurdles with developing single enantiomers with respect to synthesis, purification, and cost, we set out to identify which enantiomer of CBD3063 was responsible for the effects on N-type Ca^2+^ currents. Additionally, we evaluated the CBD3063 racemic mixture and both enantiomers in a translationally relevant model of inflammatory pain.

## 2 Material and methods

### 2.1 Animals

All procedures and protocols involving animals were approved by University of Florida’s Institutional Animal Care and Use Committee (IACUC202400000002), in accordance with the National Institutes of Health Guide for Care and Use of Laboratory Animals (Publication No. 85–23, revised 1985) and the Guidelines on Ethical Standards for Investigation of Experimental Pain in Animals*.* As there were sex-specific effects noted of CBD3063 previously (Gomez et al., 2023), only female C57BL/6J mice (12 and 4 weeks old) from the Jackson Laboratory were used for capsaicin pain model and for electrophysiological recordings, respectively. All animals were housed in acrylic cages under controlled conditions (temperature 23 °C ± 1 °C and 50 % humidity) with 12/12 light–dark cycles and tap water and food ad libitum.

### 2.2 Dorsal root ganglion neuron culture

4-week-old female C57BL/6J mice were euthanized following the guidelines of the American Veterinary Medical Association for the euthanasia of animals. In brief, lumbar and thoracic DRG were dissected and enzymatically dissociated in DMEM media (Cat. No. 11965, Thermo Fisher Scientific, Waltham, MA) containing collagenase type I (1.66 mg/mL; Cat. No. LS004194, Worthington Biochemical, Lakewood, NJ) and neural protease (1.04 mg/mL; Cat. No. LS02104, Worthington Biochemical, Lakewood, NJ) for 50 min at 37 °C with gentle agitation. Subsequently, the sensory neurons were centrifugated at 800 rpm for 5 min and resuspended in complete DRG media (DMEM supplemented with 1 % penicillin/streptomycin sulfate (Cat. No. 15140, Life Technologies, Carlsbad, CA), 10 % fetal bovine serum, and 15 ng/ml nerve growth factor (Cat. No. N2513, Millipore Sigma, St. Louis, MO). The dissociated cells were then seeded on poly-D-lysine-coated coverslips and incubated at 37 °C and 5 % CO_2_. Cultures were utilized within 24 h after seeding.

### 2.3 Whole cell patch-clamp recordings of N-type Ca^2+^ currents in dissociated neurons

Mouse DRG sensory neurons were incubated overnight either with 0.1 % DMSO (control), 20 µM CBD3063 (racemic mixture), 20 µM (R) CBD3063 or 20 µM (S) CBD3063. Twelve-millimeter round coverslips containing DRG neurons were transferred from culture medium to an external solution containing a CaV inhibitor cocktail: Nifedipine (10 µM, L-type), SNX482 (200 nM, R-type), ω-agatoxin (200 nM, P/Q-type) and Z-944 (1 µM, T-type), in order to isolate N-type Ca^2+^ calcium currents as we previously described (Tang et al., 2024). Each coverslip was kept in the recording chamber for ∼ 50 min. The external solution consisted of the following (in mM): 110 N-methyl-D-glucamine, 10 BaCl_2_, 30 TEA-Cl, 10 HEPES, 10 glucose, 0.001 TTX (pH 7.29 adjusted with TEA-OH, and mOsm/L = 310). Patch pipettes were filled with an internal solution containing (in mM): 150 CsCl_2_, 10 HEPES, 5 Mg-ATP, and 5 BAPTA, (pH 7.24 adjusted with CsOH, and mOsm/L = 305). Peak N-type Ca^2+^ current was acquired by applying 200-millisecond voltage steps from − 70 to + 60 mV in 10-mV increments from a holding potential of − 90 mV to obtain the current–voltage (I-V) relationship. Steady-state inactivation (SSI) curves were obtained by applying an H-infinity protocol that consisted of 1.5-second conditioning pre-pulses from − 100 to + 30 mV in 10-mV increments followed by a 20-millisecond test pulse to + 10 mV.

To estimate current density (pA/pF), the current amplitude was normalized to cell size (pF). Voltage-dependence of activation and inactivation curves were fitted with the Boltzmann equation. The voltage-dependence of activation curves were obtained from the I-V protocol using the equation: G = I/(V_mem_-E_rev_), where I represents the current amplitude, V_m_ is the test potential, and E_rev_ is the extrapolated reversal potential of each neuron. The conductance (G) was normalized to the maximum conductance (G_max_). Activation curves were then fitted with the Boltzmann equation:

$$\frac{G}{G_{\max}}=\frac{1}{1+\exp(\frac{V_{0.5}-V_{m}}{\kappa })}$$

Here, G is the conductance, G_max_ is the maximal channel conductance obtained from the Boltzmann fit, V_0.5_ is the voltage corresponding to half maximal activation, V_m_ is the potential and κ is a slope factor.

Inactivation curves were obtained by dividing the peak current recorded at the test pulse by the maximal current (I_max_) steady-state inactivation (SSI) was fitted with the equation:

$$\frac{I}{I_{\max}}=\frac{1}{1+\exp(\frac{V_{m}-V_{0.5}}{\kappa })}$$

Where *I* is the current, I_max_ represents the maximal current obtained from the Boltzmann fit, V_0.5_ is the potential for half-maximal inactivation of I_max_, V_m_ is the pre-pulse membrane potential, and κ is a slope factor.

Capacitive artifacts and series resistance were compensated. Recordings made from cells with greater than a 20 % shift in series resistance compensation error were excluded from the analysis. All experiments were performed at room temperature (∼23 °C). The experimenters were blinded to the conditions tested. We recorded from unstained small-diameter DRGs (ranging between 5─12 picoFarads (pF)), which correspond to C-fibers, characterized by having unmyelinated axons and known be involved in pain transmission.

### 2.4 Capsaicin pain model

Female mice were injected with either capsaicin (12 µg in 10 µL) or vehicle (80 % saline, 10 % ethanol, 10 % Tween 80) into the left footpad using a 30G syringe, while under anesthesia with isoflurane (5 % for induction and 2 % for maintenance). One hour after the capsaicin injection, mechanical allodynia was assessed using calibrated von Frey filaments.

### 2.5 Mechanical allodynia

Mice were acclimated in transparent acrylic cages on a mesh grid floor for 60 min before the experiment. Following this habituation period, von Frey filaments were used to determine the 50 % paw withdrawal threshold using the up-down method as previously described (Chaplan et al., 1994, Dixon, 1980). The 50 % withdrawal threshold was calculated using the following equation:

50 % Threshold (g) = (10^[Xf + κδ]/10000).

Here, Xf represent the value of the last von Frey filament used (in logarithmic units), κ is a correction factor derived from a calibration table based on response patterns, and δ indicates the average differences between stimuli logarithmic units (Chaplan et al., 1994).

### 2.6 Statistical analysis

Electrophysiology data were assessed for Gaussian distribution using a D’Agostino-Pearson test using GraphPad Prism 10 (GraphPad Software Inc., La Jolla, CA). Statistical significance of differences between means was determined using a one-way ANOVA test, followed by a Tukey’s multiple comparations test for peak current density and voltage-dependence of activation and inactivation parameters. For mechanical allodynia, differences were assessed using Two-Way ANOVA followed by the Tukey’s test, as independent groups were compared, using GraphPad Prism 10 (GraphPad Software Inc., La Jolla, CA). Sample size for behavioral assays was calculated with 80 % power and an expected effect size of *d* = 2.2 in behavioral experiments, with a significance level set to 0.05 using G*Power (version 3.1.9.2) based on our previous works (Gomez et al., 2023, Rodríguez-Palma et al., 2025). All values are presented as the mean ± SEM. Statistical significance was accepted at *P* < 0.05.

## 3 Results and Discussion

**Fig. 2:**
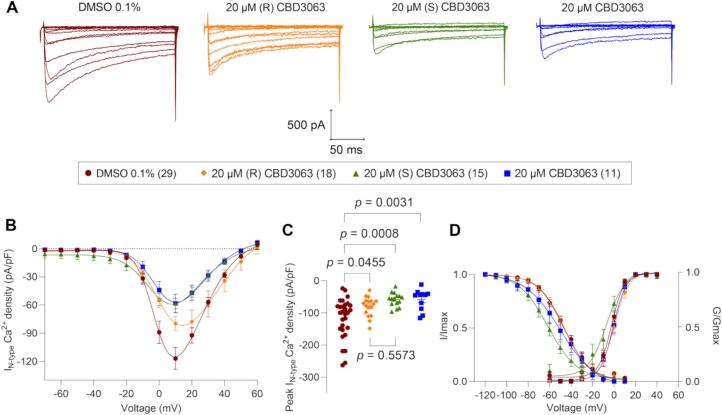
**(S) CBD3063 enantiomer inhibits N-type Ca^2+^ currents in DRG neurons to a degree comparable to the racemic mixture of CBD3063. (A)** Representative traces of N-type Ca^2+^ currents recorded from a DRG neuron under control conditions or following overnight incubation with (R) CBD3063 (20 µM), (S) CBD3063 (20 µM), or racemic CBD3063 (20 µM). **(B)** Summary of current density versus voltage (I-V) curves obtained from DRG neurons under control (0.1 % DMSO; cherry circles, n = 29), (R) CBD3063 (20 µM; yellow diamonds, n = 18), (S) CBD3063 (20 µM; green triangles, n = 15) or CBD3063 (20 µM; blue squares, n = 11). **(C)** Peak current density plot as in (B). **(D)** Voltage dependence of activation obtained from current traces as in (B), both current density and channel conductance were normalized to their corresponding maximal values (i.e., I_max_ and G_max_) that were calculated from each DRG neuron. The average maximal values (G_max_ and I_max_), and the kinetic parameters describing the corresponding voltage dependences of activation and inactivation are given in Table 1. Statistical significance of differences between means was determined using one-way ANOVA, followed by a Tukey’s multiple comparations test for peak current density and voltage-dependence of activation and inactivation parameters. Data are presented as the mean ± SEM.

To investigate the effects of CBD3063 and its enantiomers on Ca_V_2.2 channels, we performed voltage-clamp electrophysiology experiments on cultured mouse DRG sensory neurons. N-type calcium currents were isolated via acute incubation with a cocktail of specific blockers targeting the other calcium channel subtypes (i.e., R-, P/Q-, L- and T-type) expressed in these neurons. DRGs neurons were held at −90 mV and depolarized by 200-millisecond voltage steps from −70 mV to + 60 mV in 10-mV increments. Ca_V_2.2 currents were normalized to cell capacitance to obtain the current–voltage relationships shown in Fig. 2. We first evaluated the effect of the CBD3063 (20 µM; Fig. 2A-C, blue squares). Overnight incubation of CBD3063 significantly reduced the peak current of density of N-type Ca^2+^ currents by 51 % (−58.36 ± 9.86 pA/pF, Fig. 2A-C, blue squares) when compared to the control (−117 ± 11.66 pA/pF; Fig. 2A-C, DMSO 0.1 %, cherry circles). This is consistent with our previous report in rat DRG sensory neurons, where 20 µM CBD3063 (racemic mixture) reduced N-type Ca^2+^ currents by 46.5 % with respect to control (Gomez et al., 2023). Similarly, overnight incubation with (S) enantiomer (20 µM) inhibited N-type Ca^2+^ currents by 50 % (−58.43 ± 5.53 pA/pF, Fig. 2A-C, green triangles) compared to control (Fig. 2A-C). In contrast, the (R) enantiomer of CBD3063 (20 µM) reduced the peak current of density of N-type Ca^2+^ currents by ∼ 32 % (−79.70 ± 7.32 pA/pF; Fig. 2A-C, yellow diamonds) compared to control. However, no significant difference was observed between the effects of the (S) and (R) enantiomers (Fig. 2C). No changes were observed in the voltage dependence of activation between the groups. (S) enantiomer of CBD3063 induced a significant leftward shift (∼ 15 mV) in steady-state inactivation (−61.46 ± 1.77 mV, Fig. 2D, Table 1), with respect to the control (−46.64 ± 0.86 mV, Fig. 2D, Table 1). Taken together, these results suggest that both enantiomers contribute to the effect of CBD3063 on N-type Ca^2+^ currents.

**Table 1:** Biophysical properties of N-type Ca^2+^ voltage gated channels from mouse DRG neurons in the absence and presence of CBD3063 or its enantiomers.

Condition	IV curve		Activation			Fast inactivation		
	***Peak/density*** ***(pA/pF) at 10 mV***	***n***	***V_0.5_*** ***(mV)***	***k***	***n***	***V_0.5_*** ***(mV)***	***k***	***n***
0.1 % DMSO	−117.0 ± 11.66	29	−2.32 ± 0.72	5.90 ± 0.64	29	−46.64 ± 0.86	−11.96 ± 0.79	29
20 µM (R) CBD3063	−79.70 ± 7.32*	17	−0.13 ± 0.78	6.71 ± 0.69	17	−46.97 ± 1.39	−12.29 ± 1.29	17
20 µM (S) CBD3063	−58.43 ± 5.53*	15	−7.04 ± 1.76	8.38 ± 1.60	15	−61.45 ± 1.76*	−13.69 ± 1.68	15
20 µM CBD3063	−58.36 ± 9.86*	11	−0.77 ± 0.76	6.10 ± 0.68	11	−49.78 ± 2.85	−16.69 ± 2.93	11

Values are presented as means ± SEM. Gating properties were calculated from fits of the data obtained from the indicated number of individual cells to the Boltzmann equation. The values include V_0.5_, which represents the midpoint potential (mV) for voltage-dependent of activation or inactivation, and k, the slope factor. These values pertain to Fig. 1. * p < 0.05 with respect to 0.1 % DMSO.

**Fig. 3:**
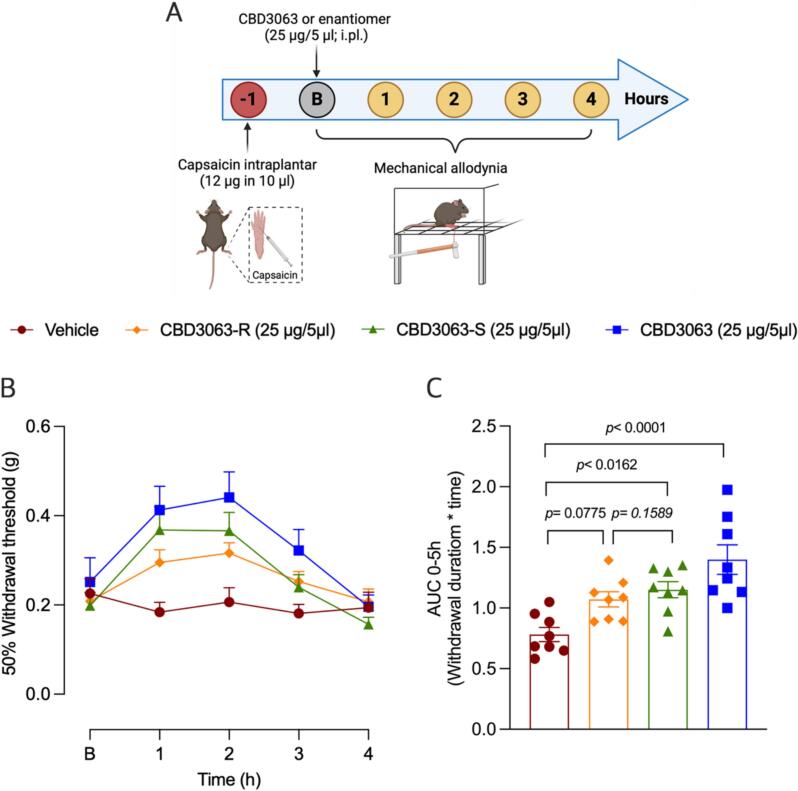
**CBD3063-S exhibit a greater antinociceptive effect compared to CBD3063-R. (A)** Timeline of experimental approaches used to assess the antinociceptive effect of racemic CBD3063, (R) CBD3063, or (S) CBD3063 in female mice with capsaicin-induced inflammatory pain. Baseline paw withdrawal thresholds were measured before the intraplantar (i.pl.) administration of racemic CBD3063 (25 µg/5 µl), (R) CBD3063 (25 µg/5 µl), (S) CBD3063 (25 µg/5 µl), or vehicle in female mice with inflammatory pain. **(B)** Time courses of the effect of intraplantar injection of racemic CBD3063, (R) CBD3063, (S) CBD3063 or vehicle on mechanical allodynia. **(C)** Quantification of area under the curve (AUC) of the time course of the effect induced by racemic CBD3063, (R) CBD3063, (S) CBD3063 on mechanical allodynia in female mice. Data are presented as the mean ± SEM (n = 8 animals per group). Statistical comparisons in panel B were performed using two-way ANOVA (time * treatment), followed by Tukey’s post hoc test. Panel C results were analyzed using one-way ANOVA, followed by the Tukey test. *P* values are indicated.

Next, we evaluated the antinociceptive effect of CBD3063 and the R and S enantiomers in vivo using the capsaicin-induced model of inflammatory pain (Rodríguez-Palma et al., 2025). This model is considered highly relevant for translational pain research, as topical capsaicin has been reliably used to test the efficacy of newly developed analgesic compounds (Wallace et al., 2016). One hour after capsaicin administration, female mice received an intraplantar injection of CBD3063 and its enantiomers and their effect on the mechanical allodynia was determined (Fig. 3A). CBD3063 injection (25 µg/5 µl i.pl.) increased the paw mechanical withdrawal threshold for 3 h in female mice, which can be interpreted as an antinociceptive effect (Fig. 3B-C). These findings align with previous observations showing that the CBD3063 is effective in reducing both neuropathic and inflammatory pain in rats and mice (Gomez et al., 2023, Allen et al., 2024). To determine which enantiomer houses the antinociceptive activity, (R) and (S) CBD3063 (25 µg/5 µl) were administrated via intraplantar injection in mice with capsaicin-induced inflammatory pain. (S) enantiomer exhibited a better antinociceptive effect compared than the effect of (R) enantiomer (Fig. 3B-C) when compared to vehicle. Moreover, the effect of (R) enantiomer (*p* = 0.0379), but not the (S) enantiomer (*p* = 0.1589), is significantly lesser when compared to CBD3063. However, direct comparison between the two enantiomers did not reveal a statistically significant difference (Fig. 3C). Taken together, these findings suggest that both enantiomers contribute to the antinociceptive properties of CBD3063, with no clear evidence that one is superior to the other. Previous studies have shown that the antinociceptive effect exhibited by racemic mixtures such as norketamine, mirtazapine, mexiletine, and MP-101 in pain models are typically mediated by a single enantiomer (Bonifacino et al., 2022, Freynhagen et al., 2006, Holtman et al., 2008, Sinnott et al., 2000). This difference may arise from variances in pharmacodynamics, pharmacokinetics, and target affinity (Brooks et al., 2011, Ceramella, 2022), which could explain the distinct effects observed between enantiomers. While we have not yet evaluated the pharmacodynamic and pharmacokinetic profile or target affinity of CBD3063 enantiomers, our electrophysiological and behaviors approaches suggest that antinociceptive effect of CBD3063 is likely to be mediated by both enantiomers.

As (R) CBD3063 reduced N-type Ca^2+^ currents by 32 % in DRG neurons and exhibited a slightly lower antinociceptive effect in vivo than the (S) enantiomer, we hypothesize that chirality is indeed a determining factor in the efficacy of CBD3063. There is evidence suggesting that chirality influences the efficacy and mechanism of actions of certain drugs (Brooks et al., 2011, Ceramella, 2022). A classic example is ibuprofen, where the (S) enantiomer is primarily responsible for its analgesic and anti-inflammatory effects (Hao et al., 2005). However, the (R) enantiomer of ibuprofen is not entirely inactive; it undergoes chiral inversion, which converts it into the active (S) ibuprofen, contributing to its overall therapeutic effect (Hao et al., 2005, Baillie et al., 1989). Whether (R) CBD3063 undergoes a similar process is unknown, but it remains a possibility that chiral inversion could potentially explain its observed effect in vitro and in vivo. A limitation of this study is that only one dose of the compounds was tested, and testing additional doses may provide a more definitive understanding of enantioselectivity.

In conclusion, our results indicate that both enantiomers (S) and (R) enantiomers of CBD3063 are active and alleviate capsaicin-induced inflammatory pain.

## CRediT authorship contribution statement

**Santiago Loya-López:** Writing – review & editing, Writing – original draft, Methodology, Investigation, Formal analysis, Data curation. **Erick J. Rodríguez-Palma:** Writing – review & editing, Writing – original draft, Visualization, Methodology, Investigation, Funding acquisition, Formal analysis, Data curation. **Aida Calderón-Rivera:** Methodology, Investigation, Formal analysis, Data curation. **Kimberly Gomez:** Methodology, Investigation, Funding acquisition, Formal analysis, Data curation. **Samantha Perez-Miller:** Writing – review & editing, Writing – original draft, Visualization, Data curation, Conceptualization. **Rajesh Khanna:** Writing – review & editing, Writing – original draft, Supervision, Resources, Project administration, Funding acquisition, Conceptualization.

## Declaration of competing interest

The authors declare that they have no known competing financial interests or personal relationships that could have appeared to influence the work reported in this paper.
